# A cerebrospinal fluid microRNA signature as biomarker for glioblastoma

**DOI:** 10.18632/oncotarget.18332

**Published:** 2017-06-01

**Authors:** Johnny C. Akers, Wei Hua, Hongying Li, Valya Ramakrishnan, Zixiao Yang, Kai Quan, Wei Zhu, Jie Li, Javier Figueroa, Brian R. Hirshman, Brittney Miller, David Piccioni, Florian Ringel, Ricardo Komotar, Karen Messer, Douglas R. Galasko, Fred Hochberg, Ying Mao, Bob S. Carter, Clark C. Chen

**Affiliations:** ^1^ Center for Theoretical and Applied Neuro-Oncology, University of California, San Diego, CA, USA; ^2^ Department of Neurosurgery, Huashan Hospital, Fudan University, Shanghai, China; ^3^ Biostatistics Department, Moores Cancer Center, UC San Diego Health System, La Jolla, CA, USA; ^4^ Department of Neurosurgery, Moores Cancer Center, UC San Diego Health System, La Jolla, CA, USA; ^5^ Department of Neurosurgery, Klinikum rechts der Isar, Technische Universität München, Munich, Germany; ^6^ Department of Neurological Surgery, Miller School of Medicine, University of Miami, Miami, FL, USA; ^7^ Department of Neurosciences, University of California, San Diego, CA, USA; ^8^ State Key Laboratory of Medical Neurobiology, Institutes of Brain Science, The Collaborative Innovation Center for Brain Science, Fudan University, Shanghai, China

**Keywords:** extracellular vesicle, CSF, liquid biopsy

## Abstract

**Purpose:**

To develop a cerebrospinal fluid (CSF) miRNA diagnostic biomarker for glioblastoma.

**Experimental Design:**

Glioblastoma tissue and matched CSF from the same patient (obtained prior to tumor manipulation) were profiled by TaqMan OpenArray^®^ Human MicroRNA Panel. CSF miRNA profiles from glioblastoma patients and controls were created from three discovery cohorts and confirmed in two validation cohorts.

**Results:**

miRNA profiles from clinical CSF correlated with those found in glioblastoma tissues. Comparison of CSF miRNA profiles between glioblastoma patients and non-brain tumor patients yielded a tumor “signature” consisting of nine miRNAs. The “signature” correlated with glioblastoma tumor volume (*p*=0.008). When prospectively applied to cisternal CSF, the sensitivity and specificity of the ‘signature’ for glioblastoma detection were 67% and 80%, respectively. For lumbar CSF, the sensitivity and specificity of the signature were 28% and 95%, respectively. Comparable results were obtained from analyses of CSF extracellular vesicles (EVs) and crude CSF.

**Conclusion:**

We report a CSF miRNA signature as a “liquid biopsy” diagnostic platform for glioblastoma.

## INTRODUCTION

Glioblastoma, defined by the World Health Organization (WHO) glioma classification as grade IV astrocytoma, is the most common form of primary brain cancer in adults [1, 2]. Diagnosis of the disease remains a clinical challenge. First, error in diagnosis occurs in up to 30% of the instances where clinical decisions are based solely upon Magnetic Resonance Imaging (MRI) [3]. As such, diagnosis of the disease requires tissue acquired through cranial surgery [4]. However, morbidity for biopsy surgical resection of glioblastoma involving eloquent regions of the cerebrum can be as high as 10% [5], with permanent neurologic injury for a subset of these patients [6]. The risk is higher for surgical resection involving eloquent cerebrum [7]. Second, a subset of brain tumor patients present with co-morbidities that prohibit consideration for surgery. Analysis of the Surveillance, Epidemiology, and End Results (SEER) registry suggests that ∼20% of all afflicted patients are medically too ill to be considered for surgery [8]. We propose that these challenges can be addressed by development of a minimally invasive “liquid biopsy” platform [9].

CSF is an appealing and accessible bio-fluid for glioblastoma “liquid biopsy”. The bio-fluid lies in close proximity to tumor tissue, often bathing tumor or its associated microenvironment [10]. The CSF can be located in the brain or its ventricles, which we termed “cisternal” CSF, or the lumbar region, which we termed “lumbar” CSF. CSF in these compartments differs in chemical compositions [11, 12], suggesting limited CSF exchange between these two anatomic compartments. Whether these differences impact their diagnostic value for glioblastoma remains an open question.

Extracellular Vesicles (EVs) are cell-secreted vesicles that range 30-2000 nm in size that mediate critical biologic functions, including cellular remodeling and intracellular communication [9]. Cancer cells exhibit increased secretion of EVs, with secreted EVs containing genetic contents reflective of the cell of origin [9, 13]. In this context, there is a growing interest in EVs derived from bio-fluids, including CSF [14], as a platform for disease diagnosis [15].

Here, we examined miRNA profiles of the CSF EVs and “crude” CSF derived from glioblastoma patients. miRNA is an attractive biomarker platform given its stability in bio-fluids [15], selective over-expression in glioblastomas [13, 16, 17], and release by tumor cells into the extracellular environment [18]. Our results support the utility of CSF miRNA profiling as a “liquid biopsy” platform for glioblastoma diagnosis.

## RESULTS

### miRNA profiling of matched glioblastoma tumor and CSF EVs in human subjects

**Figure 1:**
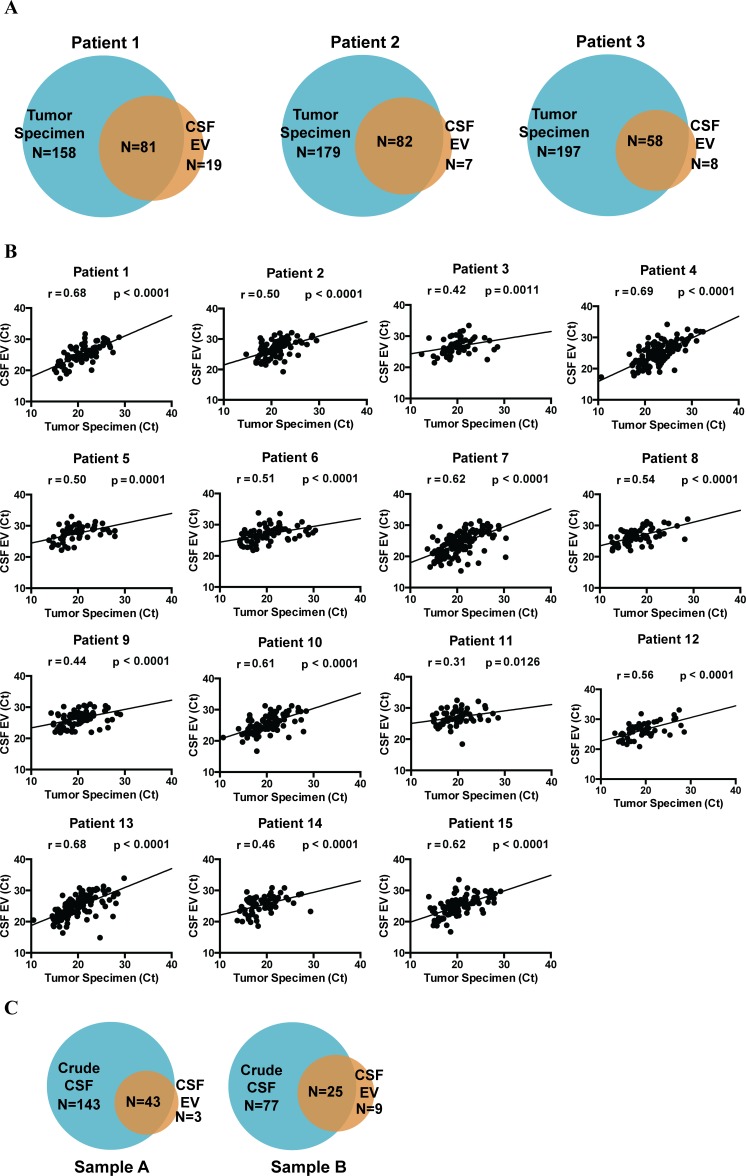
miRNA analysis of matched glioblastoma tumor and CSF samples miRNA profile of matched glioblastoma tumor and CSF EV samples were analyzed using the TaqMan OpenArray platform. **A.** Venn diagrams indicating the unique and shared detectable miRNAs between tumor tissue and CSF EVs. **B.** Correlation between miRNA profiles of matched glioblastoma specimens and CSF. For each patient, C_T_ values of shared miRNAs in tumor specimen were plotted against C_T_ values from CSF EVs. Pearson correlation coefficient was then calculated for each patient. The correlations were highly significant for all matched pairs of tumor and CSF specimens. **C.** Venn diagrams comparing the miRNA profile of crude CSF *versus* CSF EV. > 95% of miRNA found in CSF EVs were also represented in the crude CSF.

We first investigated whether the miRNA profile from CSF mirrored that of the matched glioblastoma specimen within the same subject, using the 15 subjects with matched CSF and tumor tissue from Cohort 1. Using a C_T_ cut-off of 35, we found that 200-400 miRNAs were detected in the glioblastoma specimens (median 313 species; range 238 to 351). Between 30-50% of these miRNAs were detected in the matched EV CSF (Figure 1A). However, the average C_T_ value at which these miRNAs were detected in CSF was increased by ∼5 (Supplementary Figure 1), translating to a 30-fold decrease in abundance. We plotted the level of each detectable miRNA in CSF (Figure 1B, y-axis) against its level in the glioblastoma sample (Figure 1B, x-axis) and found correlation between CSF miRNAs and tumor miRNAs for all 15 paired samples. These results suggest that the miRNA content of CSF mirrors that of matched glioblastoma samples.

**Table 1:** Patient demographics and samples

	Discovery	Discovery	Discovery	Validation	Validation
Cohort	Cohort 1 UCSD, Munich, Miami Cisternal and lumbar CSF	Cohort 2 Huashan, Lumbar CSF	Cohort 3 UCSD, Cisternal CSF	Cohort 4 UCSD, Cisternal CSF	Cohort 5 UCSD, Huashan, Lumbar CSF
**Age, Median (Range)**	61 (25-82)	59 (24-83)	56.5 (22-84)	53.5 (29-74)	58 (27-74)
**Gender**					
Female	17	32	13	5	23
Male	22	35	19	17	15
**Diagnosis**					
Glioblastoma	24	40	13	10	18
Normal/non-oncologic	15	27	19	12	20
**Collection Method**					
Cisternal	26	0	32	22	0
Lumbar	13*	67	0	0	38
**Tumor tissue**	yes	no	no	no	no

*All 13 lumber CSF samples from Cohort 1 were from the glioblastoma group

### Comparison of CSF fractions for number of miRNA species

For select samples, miRNA profiling was performed for both CSF derived EV and crude CSF. In general, more miRNA species were detected in the crude CSF relative to EV. Nearly all miRNAs detected in the EVs were also present in the crude CSF (Figure 1C).

### Identification of a miRNA CSF signature which can identify glioblastoma

Though all CSF samples were collected using the same Standard Operating Procedure (SOP), significant variation in miRNA profiles were found between CSF derived from the first three cohorts (cohorts 1, 2, and 3). To account for this variability, we used miRNA profiles derived from all three cohorts in our signature development. Details of the analysis can be found in Supplementary Figure 2. In brief, miRNAs with levels that differed between glioblastoma and non-oncologic CSF were identified using the criteria of FDR < 0.2 and log(fold-change) > 2 as described. From Cohort 1, we identified 29 miRNAs. 3 miRNAs were identified in Cohort 2. In Cohort 3, we identified 110 miRNAs as differentially expressed, with miR-21 having the largest fold change as previously published [16]. Based on our cross-sample validation criteria, 24 miRNAs were subsequently selected for signature development (Supplementary Figure 3). In addition, three differentially regulated miRNAs which validated in one (but not two) independent datasets were added to the candidate set. miR-548a, stably expressed across the three data sets and potentially useful as a reference miRNA, was also added to the panel, yielding a total of 28 candidate miRNAs.

**Figure 2:**
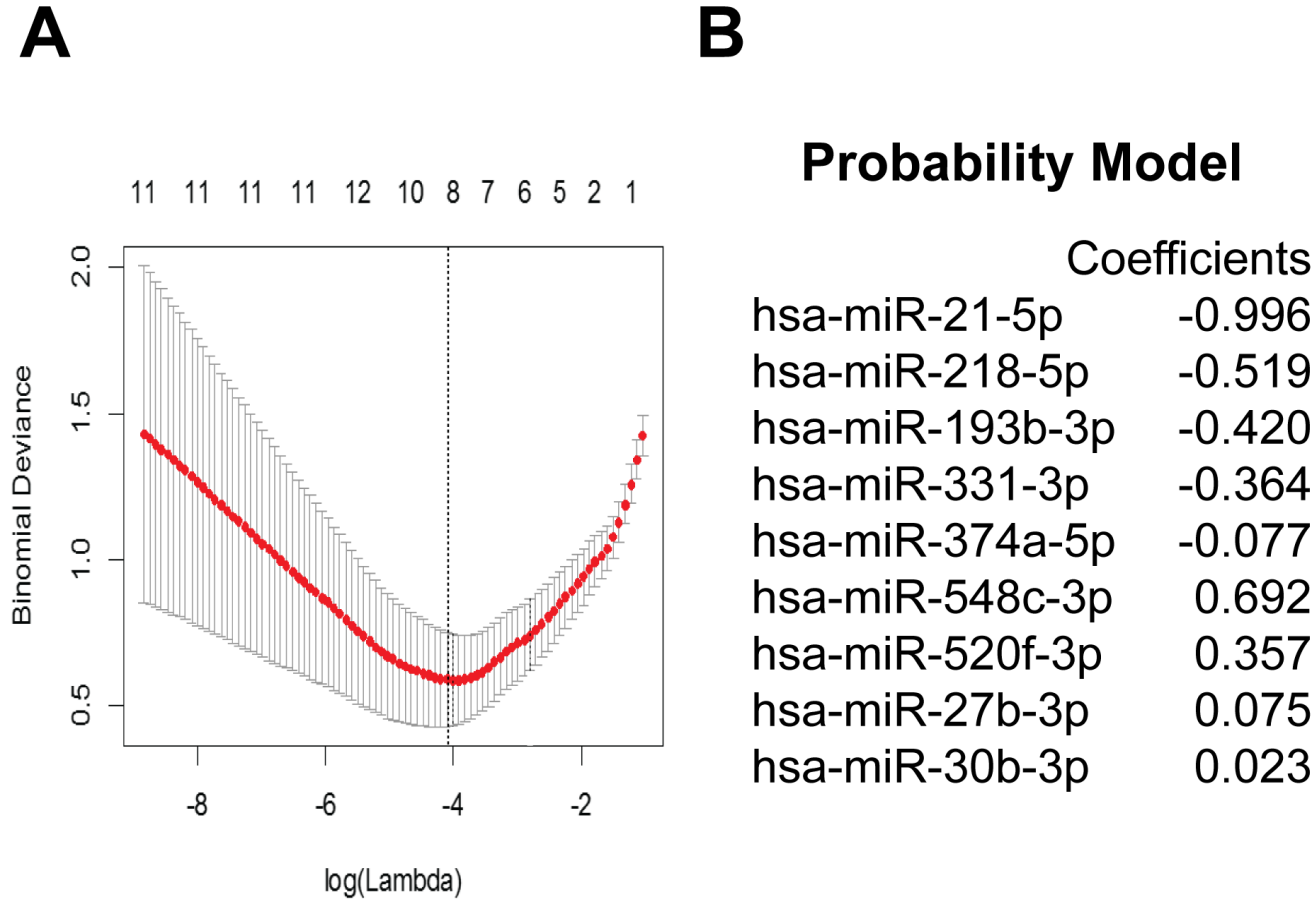
Identification of miRNA signature Differentially expressed miRNAs between glioblastoma and non-oncologic CSF samples were selected from miRNA qPCR array based on FDR < 2 and log(fold-change) > 2 and cross-validated using multiple cohorts. **A.** 28 candidate miRNAs was used to train a classifier with LASSO using a using cross-validated minimum deviance as the model selection criterion, **B.** yielding a 9 miRNA signature.

We then used LASSO [19] to develop a classifier from these 28 candidate miRNAs using cross-validated minimum deviance as the model selection criterion (Figure 2A). LASSO analysis indicated an optimal classifier consisting of 9 miRNAs, including 5 miRNAs that were enriched (miR-21, -218, -193b, -331, and -374a) and 4 miRNAs that were depleted (miR-548c, -520f, 27b, and 130b) in glioblastoma CSF (Figure 2B). We then determined the optimal score cutoff (0.4) below which we classified a subject as non-glioblastoma and above which we classified a subject as with a diagnosis of glioblastoma. Both the signature coefficients and the cutoff for classification as glioblastoma were documented before proceeding to the validation step.

### Correlation of the CSF miRNA signature score with tumor volume

**Figure 3:**
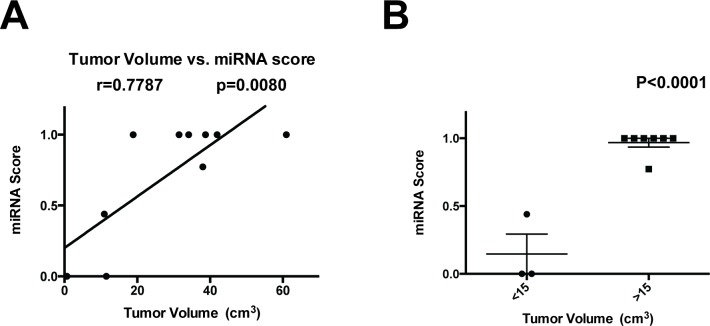
Correlation of miRNA score with tumor volume **A.** The tumor volume of 11 patients in Cohort 3 was plotted against the CSF miRNA signature score, and the Pearson correlation coefficient was calculated. **B.** Glioblastoma < 15 cc's in volume showed a lowered miRNA signature score relative to those with > 15cc’s.

Pre-operative MRI was available for 11 of the patients in Cohort 1. We created tumor volumes based on the Agfa CD Viewer and related these to the CSF miRNA gene signature scores. A positive correlation was observed between miRNA signatures and tumor volumes (Figure 3A). Glioblastoma with volumes < 15 cc had lower miRNA scores than those with > 15cc's (*P* < 0.0001, Figure 3B).

### Validation of the CSF miRNA glioblastoma signature

**Figure 4:**
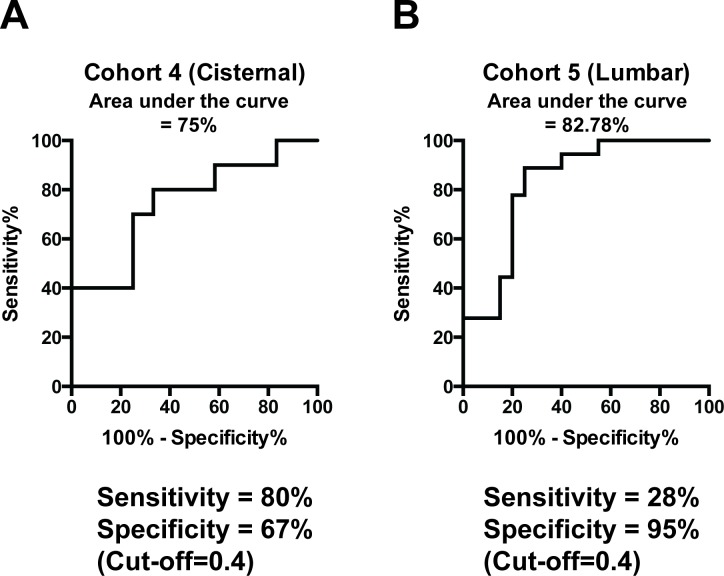
Validation of miRNA signature **A.** Performance of the 9-miRNA signature using crude cisternal CSF from an independent collection of prospectively collected samples. **B.** Performance of the 9-miRNA signature using crude lumbar CSF from an independent collection of prospectively collected samples.

We tested the performance of the 9-miRNA signature in a prospective manner. Since most EV miRNAs are also detected in crude CSF, we opted to validate our signature using unfractionated crude CSF. We prospectively collected and profiled cisternal CSF from an additional 22 patients (Cohort 4: 10 glioblastoma and 12 non-oncologic patients). Using the cutoff (0.4) established during the discovery process, the signature correctly identified 8/10 subjects with glioblastoma and 8/12 non-oncologic subjects, yielding a sensitivity of 80% and specificity of 67%. The AUC was 0.75 (95% CI 0.53, 0.97) (Figure 4A).

We also prospectively collected and profiled lumbar CSF from 18 glioblastoma and 20 non-oncologic patients (Cohort 5). Using the same coefficients and cutoff score, the 9 miRNA signature correctly identified 5/18 glioblastoma subjects and 19/20 non-oncologic subjects, yielding a sensitivity of 28% and specificity of 95%. The AUC was 83% (95% CI: 69%, 96%). (Figure 4B). Notably, few miRNA species were detected in the lumbar CSF samples. These results suggest that cisternal and lumbar CSF may differ in miRNA content. Notably, these validation samples used whole CSF for the miRNA assay, as described in methods.

13 lumbar glioblastoma CSF samples were collected as a part of Cohort 1. We had compared the performance of our miRNA signature in these samples in order to afford direct comparison to that seen in the cisternal samples. In the cohort 1 lumbar CSF samples, the 9 miRNA signature correctly identified glioblastoma subjects in 3/13 glioblastoma samples yielding a sensitivity of 23%. These results were comparable to those observed in the validation cohorts, confirming our observation that the diagnostic utility of the 9-miRNA signature is optimal when applied to cisternal CSF (Supplementary Figure 4).

### Validation of increased miR21 in a mouse xenograft model of glioblastoma

**Figure 5:**
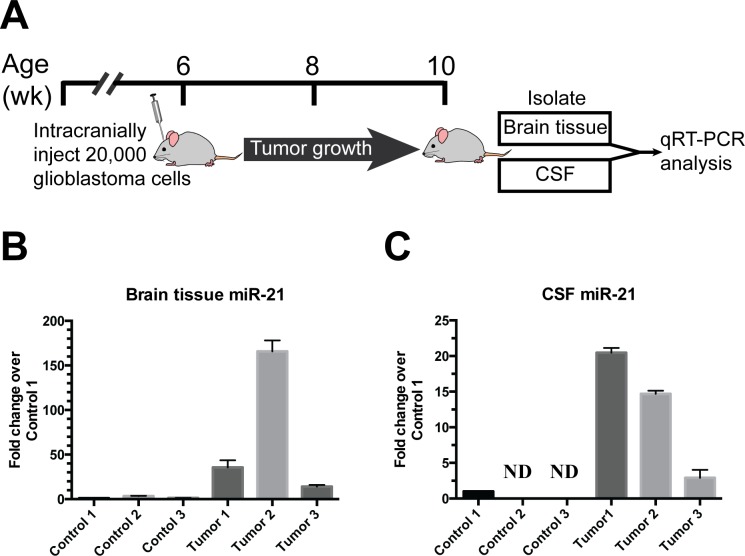
Direct release of miR-21 from glioblastoma xenograft *in vivo* **A.** 20,000 human glioblastoma stem cells were intracranially injected into nude mice. 4 weeks later, brain tissues and murine CSFs were collected from tumor bearing mice and age-matched nude mice without the xenograft injection. **B.** Human miR-21 levels were elevated in the brain tissue of patient derived glioblastoma xenograft bearing mice and undetectable in mice without xenograft implant. **C.** Human miR-21 levels were elevated in the CSF of patient derived glioblastoma xenograft bearing mice and undetectable in mice without xenograft implant.

miR-21 [16] play a pivotal role in our signature. We wished to determine whether glioblastoma growth induce accumulation of miR-21 in the CSF and used a murine xenograft model to achieve this end. We orthotopically implanted the patient-derived glioblastoma neurosphere line (JVJ), which expressed high levels of miR-21, into nude mice. 4 weeks after injection, brain tissue and murine CSF were collected from tumor bearing mice and age-matched, mock injected nude mice (Figure 5A). Both brain tissue and CSF were analyzed by qRT-PCR to measure the level of miR-21. In all analyzed samples, we found elevated miR-21 levels in the brain tissues (Figure 5B) and CSFs (Figure 5C) isolated from xenograft bearing mice relative to control mice. This result suggest that glioblastoma xenograft growth induce accumulation of miR-21 in murine CSF.

## DISCUSSION

In current clinical practice, CSF sampling is not routinely performed in glioblastoma patients. The sensitivity of CSF cytology as a diagnostic tool for glioblastoma is ∼10% [20] and below the threshold for clinical utility. However, our study suggests potential utility for CSF miRNA profiling as a diagnostic platform for glioblastoma. The miRNA detectable in human and nude mouse glioblastoma specimens is detected in matched CSF, though at a concentration that is ∼30 fold lower. miRNA profiles of CSF derived from glioblastoma patients correlated well to the miRNA profiles of the matched tumor specimens. We developed a nine miRNA CSF signature that discriminated CSF of glioblastoma patients from those of patients without history of brain cancer. We validated this signature using prospectively collected CSF samples after development and documentation of the original signature. For crude CSF based assay, the sensitivity and specificity for glioblastoma detection were 80% and 67%, respectively. In contrast, for CSF derived from lumbar puncture, the sensitivity and specificity for glioblastoma detection were 28% and 95%, respectively. It is important to note that the miRNA reported here differ from those previously reported to discriminate between types of brain cancer [21], suggesting that our miRNA signature has limited utility in discriminating between different forms of brain cancers. These results suggest that distinct miRNA profiles may be required to address different clinical needs.

There has been significant variability in the reported miRNA profiles in CSF derived from glioblastoma patients [21–23]. We observed this variability in our own study, where significant variation in miRNA profiles were found between CSF derived from the three discovery cohorts (Supplemental Figure 3). A major source of variability is the CSF collection site (cisternal *vs*. lumbar). However, even after correcting for site of collection, this variability remained. It is worthwhile noting that the CSF samples were collected in our study through a Standard Operating Procedure (SOP) and processed identically post-collection. The variability observed between these cohorts, in the context of the published literature, suggests that CSF miRNA profiles are likely influenced by physiologic factors or perturbation that was not accounted for by the SOP (e.g. circadian rhythm, fatigue, intake of medicine… etc). As such, the robustness of the CSF miRNA signatures are largely a function of the sample size, since larger sample sizes afford a greater likelihood of minimizing the undue influence of any particular perturbation/physiology. Our study is particularly important in this context, since our study design is the only one in the literature that derived the signature through three independent cohorts, summing to 135 CSF samples. We subsequently validated our results in another 60 prospectively collected CSF. The scale of our study as well as the meticulous effort devoted to validation is notable in the reported literature of glioblastoma CSF biomarkers.

An important finding in this study is that the miRNA contents of cisternal and lumbar CSF differ. We found that less than half of the miRNAs detected in cisternal CSF were detected in lumbar CSF (Supplementary Figure 5), likely accounting for the fewer number of differentially expressed miRNAs found in cohort 2. This finding suggests the two CSF compartments do not communicate sufficiently for full equilibrium of miRNA contents. Similar observations have been made for other proteins and metabolites [11, 24]. For instance, IgG level decreases progressive as the CSF moves from the site of intracranial inflammation to the lumbar sac [25]. These differences bear relevance to CSF based diagnostics and warrant consideration in future study design. For instance, separate miRNA signatures may need to be developed for analysis of clinical lumbar and cisternal CSF samples.

EVs have been touted as platforms for diagnostic and prognostic biomarker interrogation [26–28]. The isolation of these EVs from CSF introduces an additional step during clinical sample processing [29], a step which incurs increased cost and risk of contamination risk. The step is necessary if 1) the biomarker of interest is enriched in EVs or 2) if inhibitory factors prohibitive to the analytical platform is present in the crude CSF. Our analysis support neither hypothetical scenarios. When we compared the miRNA profiles of CSF EVs relative to crude CSF, we found that > 95% of miRNAs found in EVs (including miRNAs in our signature) were represented in the crude CSF, suggesting that crude CSF may suffice for miRNA profiling. Further supporting this hypothesis, the performance of the 9 miRNA signature was comparable when applied to CSF EV RNA (Supplementary Figure 6) or crude CSF RNA (Figure 4).

The literature that examined altered miRNA regulation in glioblastoma has expanded over the past decade [30]. It is notable that of the reported miRNA that are significantly over- or under-expressed in clinical glioblastoma specimens [30], only miR-21 was represented in our miRNA signature. As further validation of our correlative clinical studies, we showed that murine CSF miR-21 levels were elevated in murine CSF from glioblastoma xenograft bearing mice (Figure 5). We did not observe such increase for other miRNAs previously reported to be over-expressed in glioblastoma, including miR-16 [30–32] or miR-10b [17, 30, 33] (data not shown). Our previous study suggested that > 90% extra-cellular miR-21 were found in the EV fractions [13]. Together, these results suggest that glioblastoma harbor biologic mechanisms that facilitate the exportation of miR-21 through EV secretion. This interesting hypothesis awaits experimental validation.

While our miRNA signature performed well as a diagnostic tool in cisternal CSF, opportunities for obtaining these samples are admittedly limited. Such samples can be obtained only from patients with an Ommaya reservoir or a ventriculo-peritoneal shunt. Because these procedures involve placement of an indwelling catheter that is in direct communication to cisternal CSF, serial samples can be safely acquired in this patient population. As such, clinical testing of the cisternal CSF signature is feasible in the subpopulation of glioblastoma patients with an indwelling shunt system. Moreover, serial sampling of cisternal CSF from this patient population may afford a minimally invasive platform for tracking glioblastoma disease burden. We are in the process of collecting and testing CSF from recurrent glioblastoma patients to further test the utility of our miRNA signature.

In sum, our study provides a proof-of-principle study demonstrating the plausibility of CSF miRNA profiling as a “liquid biopsy” platform for glioblastoma diagnosis and provides the basis of future validation of this platform.

## MATERIALS AND METHODS

### Clinical specimen collection and image analysis

Five cohorts of patients totaling 195 subjects provided CSF for these studies (Table 1). The CSF studies were approved by IRB boards at University of California San Diego (UCSD) (Cohorts 1, 3, 4, and 5), Technische Universität München (TUM)(Cohorts 1), University of Miami Hospital (UMH)(Cohorts 1), and Huashan Hospital(Cohorts 2,5). All studies were in conducted in accordance with the principles expressed at the declaration at Helsinki. Each patient was consented in writing by a research coordinator prior to CSF collection. Median age ranged from 54 to 61 years across cohorts. Overall, 88 subjects were female and 107 were male, 111 had diagnosis of glioblastoma and 84 had other non-oncologic conditions. Cisternal and ventricular CSF (grouped as “cisternal”) was collected on 80 subjects by drain placement or cisternal aspiration at the time of craniotomy prior to tumor manipulation. Lumbar CSF was collected on 115 subjects, through lumbar puncture or lumbar drain. Collected CSF specimens were filtered (0.8μm filter), immediately frozen and stored at −80°C. 1 cc of CSF was utilized as the input for all miRNA analysis. The UCSD cohort was additionally consented for analysis of MR images. Volumetric measurements of available pre-operative MR images were carried out with Agfa CD Viewer 4.5.1 using the formula Volume = (L × W × H)/2, where L is the greatest length, W is the greatest width, and H is the greatest depth or height of the tumor [34]. Patients that received bevacizumab were excluded from MR image analysis [35].

### Extracellular vesicle (EV) isolation

The EV fraction was isolated by differential centrifugation as previously described [13]. CSFs were diluted 1:1 with 1x PBS (Mediatech) prior to centrifugation. Samples were centrifuged at 2,000×g for 20 min to remove cellular debris. The supernatant was further centrifuged at 120,000×g for 2 h in a Type 70 Ti rotor (Beckman) to pellet the EVs. All centrifugation steps were performed at 4°C. EV pellets were resuspended in PBS and stored at −80°C.

### miRNA profiling

RNA was extracted from each sample using the miRCURY™ RNA Isolation Kit (Exiqon). Samples assayed were EV, supernatant and tissue from cohort 1; EV and supernatant from cohort 2; EV from cohort 3; and whole CSF from cohorts 4 and 5. Four microliters of RNA extract (4-20ng/μl) was used as input for microRNA profiling on the TaqMan^®^ OpenArray^®^ Real-Time PCR System using the manufacturer's instructions (Life Technologies). Manufacturer's cartridges consisted of 818 TaqMan qPCR assays arranged on 384 well plates, with primers targeting 754 miRNA species, and 16 replicate wells of one negative and 3 positive RNA controls. Megaplex™ RT Primers, Human Pool A v2.1 and Megaplex™ RT Primers, Human Pool B v3.0 were used for the reverse transcription step. Megaplex™ PreAmp Primers, Human Pool A v.2.1 and Megaplex™ PreAmp Primers, Human Pool B v3.0 were used for the PreAmp step. The samples within each of the 3 discovery cohorts were assayed on the same date using the same reagents. The validation samples (cohorts 4 and 5) were assayed in two different batches on two different dates and data was combined for analysis.

### Data normalization, QC, and preprocessing

miRNA species with C_T_ value ≥35 were considered below the detection threshold. In tumor tissue samples, C_T_ values for the query miRNAs were normalized using the mean of the positive controls (RNU44, RNU48, U6-rRNA). For the CSF samples, the positive control miRNAs were not uniformly expressed at high levels across samples. For the discovery cohorts global mean normalization was performed in which normalized C_T_ values were calculated as the raw C_T_ value minus the arithmetic mean of all expressed miRNAs in the sample [36]. For the validation cohorts, the data was first normalized within each sample as before using the global mean normalization. Then the batch effect from the two assay dates was removed using an empirical Bayes approach (ComBat) [37] with assay date and two confounding variables (pathology and CSF collection site) included in the adjustment model. The batch-corrected data were then combined for analysis.

### Statistical approach to training and validation of the classifier

The classifier was trained using the three discovery cohorts (Cohorts 1, 2, and 3). When both supernatant and EV miRNA was available within a cohort, we used the fraction with the higher median number of detected miRNA species for analysis within that cohort. For cohorts with low detection rates, we used both fractions with a Bonferroni correction for the two comparisons. Differentially expressed CSF miRNAs between glioblastoma and non-oncologic subjects were identified using the limma Bioconductor package [38], with FDR < 0.2 and log (fold-change) > 2 as criteria. We then required candidate miRNAs from a given cohort to replicate as differentially expressed in at least 2 additional discovery data sets, including Cohort 1 EV, Cohort 2 EV + supernatant, Cohort 3 EV and also including tissue miRNA data from TCGA [39]. The replication criterion was a two-sided *p*-value < 0.05 (from limma, or a *t*-test for TCGA) and the same direction of differential expression; this test of replication has overall Type I error rate ∼1%. This candidate selection plan was pre-specified and documented.

Candidate miRNAs were carried forward to a multivariate model to discriminate glioblastoma from non-oncologic controls using L1-penalized logistic regression [19]. The model was trained with Cohort 3 using glmnet package in R with lambda chosen by cross validation [19]. The signature and optimal cutoff score to discriminate cases from controls in Cohort 3 were documented. The prediction error of the classifier with pre-determined cutoff was then evaluated data from whole CSF, using the prospectively collected independent validation Cohorts 4 and 5. For correlation analyses, the Pearson correlation coefficient was calculated using Graphpad Prism 6.

### Orthotopic xenograft model

Dissociated glioblastoma stem cells JVJ (2×10^4^ cells in 4 μl HBSS) were stereotactically injected into the brains of nude mice at age 6 weeks old. The coordinates were: 1.8 mm to the right of bregma and 3 mm deep from the dura. Aged-matched nude mice were used as controls. Four weeks after injection, CSF samples were collected from the cisterna magna as previously described [40]. Mock injection with vehicle control was carried out for control mice.

### Quantitative reverse transcriptase-polymerase chain reaction (qRT-PCR)

For the detection of tissue miRNA, RNA was extracted from homogenized mouse brains using Qiagen miRNeasy Mini Kit. cDNA was synthesized using TaqMan miRNA Reverse Transcription Kit and miRNA-specific stem-loop primers (Applied Biosystems), followed by qPCR using SsoAdvanced™ Universal Probes Supermix (Bio-Rad) and miRNA specific Taqman assay on a Bio-Rad CFX96 instrument. For the detection of miRNA from murine CSF, collected CSF was lysed directly in buffer containing 50mM Tris pH 8, 140mM NaCl, 1.5mM MgCl_2_, 0.5% NP40, and 0.1% BSA, then reverse transcribed using SuperScript^®^ VILO™ cDNA synthesis kit. The cDNA was pre-amplified for 15 cycles using Taqman PreAmp Mastermix prior to PCR detection with miR-21 Taqman assay.

### Primer sequences

Taqman miRNA assay for miR-10b, miR-16, and miR-21 were purchased from ThermoFisher Scientifics.

## SUPPLEMENTARY MATERIALS FIGURES


