# Supplementary material for: A cerebrospinal fluid microRNA signature as biomarker for glioblastoma
Source: Oncotarget. 2017 Jun 1;8(40):68769–79. doi: 10.18632/oncotarget.18332 (PMC5620295; doi:10.18632/oncotarget.18332)
Supplement: Supplementary file 1 [file oncotarget-08-68769-s001.pdf]

A cerebrospinal fluid microRNA signature as biomarker for glioblastoma

SUPPLEMENTARY MATERIALS

Supplementary Methods

Clinical specimen collection

Fifteen pairs of matched tumor specimen and CSF were collected and used for correlation studies. These samples were collected at UMH (n=10), TUM (n=4), and UCSD (n=1). For miRNA signature development, 5 patients cohorts were collected. Cohort 1 included cisternal and lumbar CSF collected from 21 glioblastoma patients (6 from UCSD and 15 from TUM) and 15 non-oncologic controls from UCSD (trauma (n=4), subarachnoid hemorrhage (n=6), normal pressure hydrocephalus (n=3), Middle Cerebral Artery Stroke (n=1) and Intracerebral hemorrhage (n=1)). Cohort 2 included lumbar CSF collected from 40 glioblastoma patients and 27 non-oncologic controls (normal pressure hydrocephalus (n=18)) patients from Huashan Hospital in China. For Cohort 1 and Cohort 2, both EV and supernatant miRNA were profiled. Cohort 3 was comprised of 13 glioblastoma samples and 19 non-oncologic controls from UCSD (trauma (n=5), subarachnoid hemorrhage (n=8), normal pressure hydrocephalus (n=2), arteriovenous malformation (n=2), Middle Cerebral Artery Stroke (n=1) and Intracerebral hemorrhage (n=1)). Cisternal CSF was obtained from these patients, and the EV miRNA profile was determined. Cohort 4 included cisternal CSF collected from 10 glioblastoma and 12 non-oncologic patients at UCSD (trauma (n=1), subarachnoid hemorrhage (n=2), normal pressure hydrocephalus (n=4), intraparenchymal hemorrhage (n=3), intracerebral hemorrhage (n=1), and seizure (n=1)). Cohort 5 consisted of lumbar CSF collected from 18 Huashan Hospital patients and 20 healthy controls from UCSD. Fro Cohort 4 and Cohort 5, total RNA was isolated from crude CSF and profiled for miRNA expression.

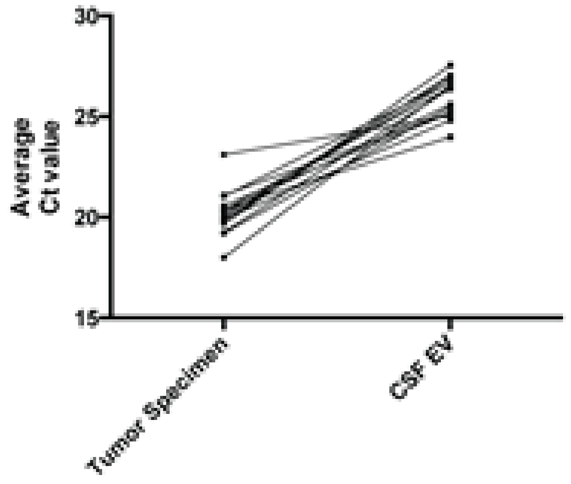

Supplementary Figure 1: Average C_T value of detectable miRNA in glioblastoma tumor specimen vs. CSF EV

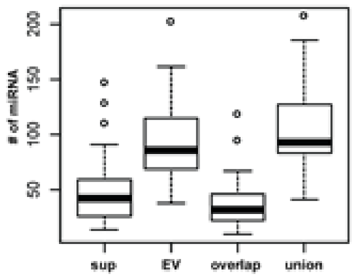

Supplementary Figure 2: Comparison of the number of miRNA species detected in the different fractions of CSF from Cohort 1.

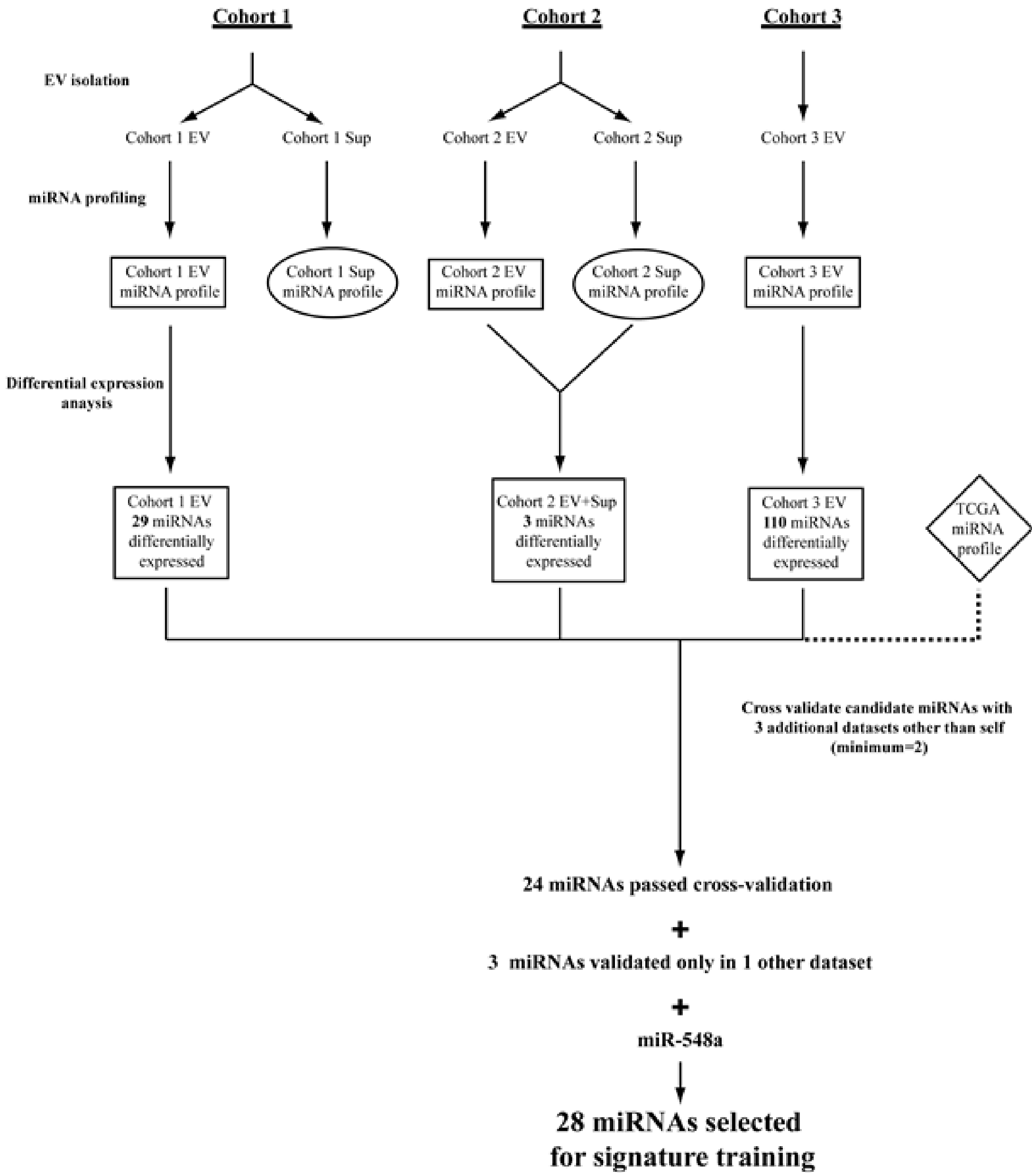

Supplementary Figure 3: miRNA profiling and candidate miRNA selection plan.

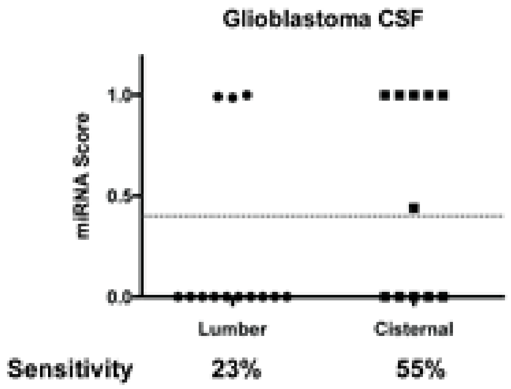

Supplementary Figure 4: Comparing the sensitivity of the 9-miRNA signature using CSF EVs isolated from lumbar and cisternal CSF (Cohort 1).

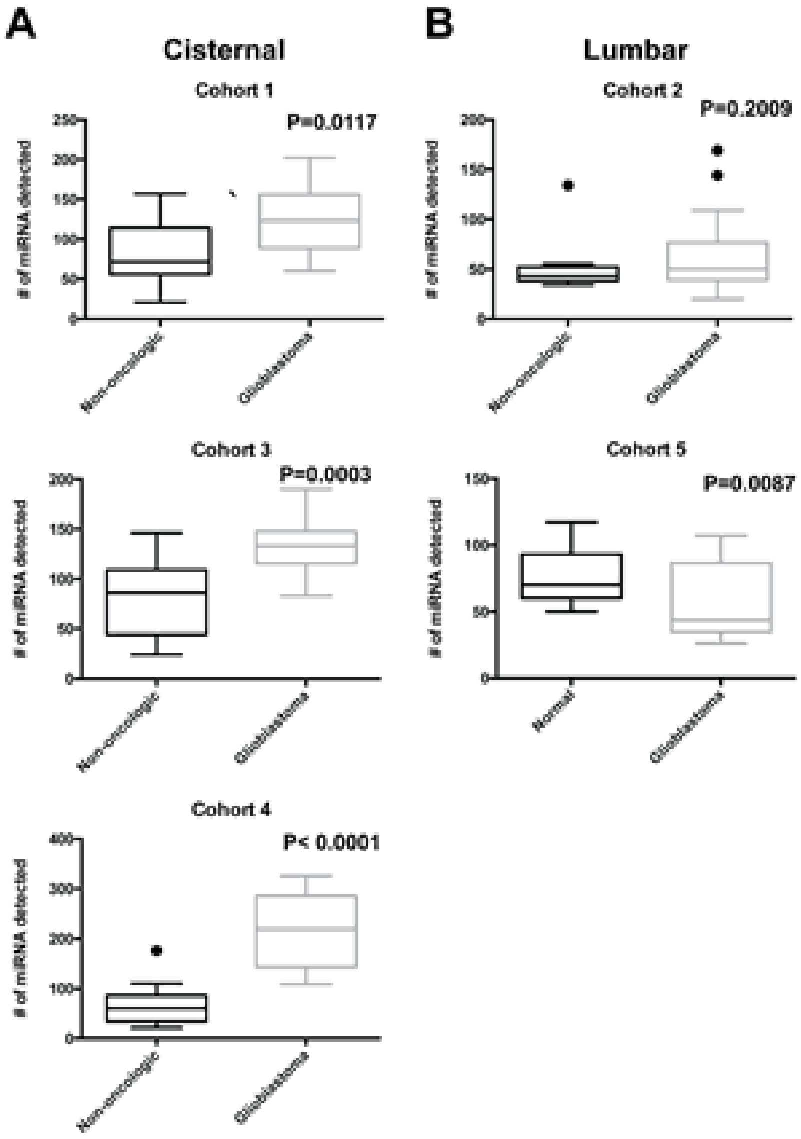

Supplementary Figure 5: Detectable number of miRNA species in (A) cisternal CSF and (B) lumbar CSF.

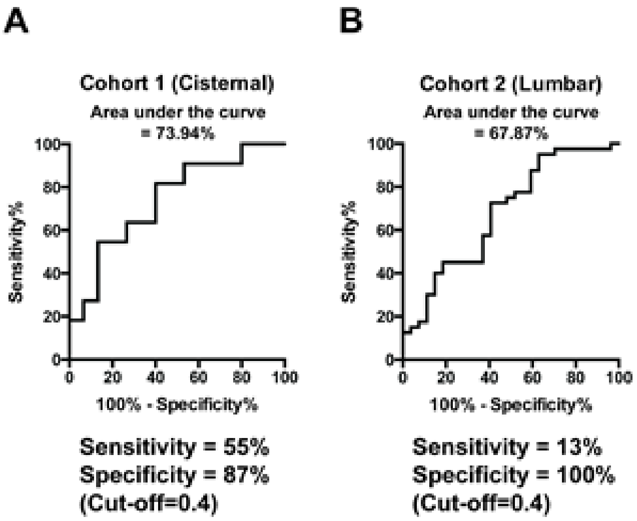

Supplementary Figure 6: (A) Performance of the 9-miRNA signature using CSF EVs isolated from cisternal CSF: Cohort 1. (B) Performance of the 9-miRNA signature using CSF EVs isolated from lumbar CSF: Cohort 2.
